# Brain Network Alterations in Rectal Cancer Survivors With Depression Tendency: Evaluation With Multimodal Magnetic Resonance Imaging

**DOI:** 10.3389/fneur.2022.791298

**Published:** 2022-06-29

**Authors:** Wenwen Zhang, Ying Zou, Feng Zhao, Yongqing Yang, Ning Mao, Yuan Li, Gang Huang, Zhijun Yao, Bin Hu

**Affiliations:** ^1^Department of Radiology, Gansu Provincial Hospital, Lanzhou, China; ^2^Department of Information Engineering, Yantai Vocational College, Yantai, China; ^3^School of Computer Science and Technology, Shandong Technology and Business University, Yantai, China; ^4^School of Management Science and Engineering, Shandong Technology and Business University, Yantai, China; ^5^Department of Radiology, Yantai Yuhuangding Hospital, Yantai, China; ^6^Big data and Artificial Intelligence Laboratory, Yantai Yuhuangding Hospital, Qingdao University, Yantai, China; ^7^School of Information Science and Engineering, Lanzhou University, Lanzhou, China; ^8^Gansu Provincial Key Laboratory of Wearable Computing, School of Information Science and Engineering, Lanzhou University, Lanzhou, China; ^9^CAS Center for Excellence in Brain Science and Intelligence Technology, Shanghai Institutes for Biological Sciences, Chinese Academy of Sciences, Shanghai, China; ^10^Beijing Institute for Brain Disorders, Capital Medical University, Beijing, China

**Keywords:** rectal cancer, depression tendency, functional network, structural network, multimodal research

## Abstract

Surgery and chemotherapy may increase depression tendency in patients with rectal cancer (RC). Nevertheless, few comprehensive studies are conducted on alterations of brain network induced by depression tendency in patients with RC. Resting-state functional magnetic resonance imaging (rs-fMRI) and diffusion tensor imaging (DTI) data were collected from 42 patients with RC with surgery and chemotherapy and 38 healthy controls (HCs). Functional network (FN) was constructed from extracting average time courses in brain regions, and structural network (SN) was established by deterministic tractography. Graph theoretical analysis was used to calculate network properties. Networks resilient of two networks were assessed. Clinical correlation analysis was explored between altered network parameters and Hamilton depression scale (HAMD) score. This study revealed impaired FN and SN at both local and global levels and changed nodal efficiency and abnormal small-worldness property in patients with RC. On the whole, all FNs are more robust than SN. Moreover, compared with HC, patients with RC show less robustness in both networks. Regions with decreased nodal efficiency were associated with HAMD score. These cognitive dysfunctions are mainly attributable to depression-related brain functional and structural network alterations. Brain network reorganization is to prevent patients with RC from more serious depression after surgery and chemotherapy.

## Introduction

Rectal cancer (RC) is a disease characterized by a high mortality rate. Patients with RC usually suffer from tremendous psychological stress, which leads to a series of psychological diseases (1, 2). Some contemporary studies showed that various discomfort symptoms caused by chemotherapy could induce cognitive impairment in patients with cancer, including impaired attention, memory, and executive function (3, 4). The long-term emotional distress of the patient increased the risk of depression (5). Depression-related factors may contribute to the less optimal network topology in the functional network (FN) (6, 7) and structural network (SN) (8) of patients with cancer. Neuroimaging studies reported that a considerable number of patients with cancer with surgery and chemotherapy had morphological variation and functional abnormalities in brain, such as decreased hippocampal volume (9), lower white matter volume (10), memory difficulties (1), and cognitive deficit (11). Therefore, research on FN and SN in this study could bring new insights into the neurophysiological mechanisms in patients with RC, and through the analysis of brain images, the emotional distress of patients with RC could be diagnosed and treated, thereby improving the quality of life of patients (12).

Combining multimodal data can reveal hidden relationships among different data, unifying different findings in brain imaging (13). Therefore, multimodal imaging is a prominent method in cognitive neuroscience research. Graph-based functional and structural brain connectivity analysis is a new method, which provides evidence for the complexity of the brain by modeling the interactions between different brain regions (14, 15). Previous studies have consistently shown that the brain functional network is organized in a small-worldness property, with local specialization and high global information transmission capabilities (16, 17). Bruno et al. (1) reported significantly reduced shortest path length and small-worldness property in the breast cancer group. Several findings point that the functional network of patients with cancer loses its ability to support various cognitive functions following chemotherapy (18, 19). Observational studies found that alterations in brain structural network had an adverse impact on the cognition of cancer survivors (20, 21). Although there are many studies on brain cognitive impairment in patients with cancer, less is known about FN and SN abnormalities in patients with RC. We used multimodal neuroimaging to investigate the alterations in FCs and SCs for gaining insight into the brain cognitive dysfunction in patients with RC.

In view of the poor understanding of psychological disorders and cognitive impairment of RC survivors in existing studies, it is essential to study the depression tendency and related factors in patients with RC. Therefore, this study investigated the abnormalities in FN and SN using graph theory analysis in patients with RC with surgery and chemotherapy characterized by depression tendency compared with healthy controls (HCs). We hypothesized that patients with RC would show altered small-worldness property and topological architecture in the FN and SN due to the effects of depression tendency. We sought to expand our understanding of the resilience of the brain network in patients with RC. The study also explored the potential association between the significant alterations in network properties of patients with RC and severity of depression symptoms.

## Methods

### Participants

A total of 42 patients with RC with surgery and chemotherapy were recruited from the Gansu Provincial Hospital, whereas the 38 age- and gender-matched healthy control participants were recruited through newspaper advertisements. They were recruited from July 2017 to June 2020. All participants were diagnosed according to DSM-IV criteria by two experienced psychiatrists. They have executed the evaluation of 17-item Hamilton Rating Scale for Depression (HAMD-17). The evaluation results showed that all the patients with RC included in the experiment had depression tendency. All participants were given written informed consent when image scanning. None of the subjects took any psychotropic drugs.

### Data Acquisition

Magnetic resonance imaging data were acquired using a 3.0 T Siemens Trio scanner (Siemens Erlangen, Germany). Subjects were asked to relax with eyes closed and not to think about anything. The structural image was acquired with a T1-weighted spin-echo sequence: TR/TE = 2530/2.98 ms, slice thickness = 1 mm, slice gap = 0.8 mm, FOV = 256^*^256 mm, The resting-state functional images (rs-fMRI) were obtained with the following parameters: TR/TE = 2,000/30 ms, 64^*^64 matrix, FOV = 224^*^224 mm, total 240 volumes, 32 sequential ascending axial slices of 3.5 mm thickness. Diffusion tensor imaging (DTI) data were acquired using a single-shot echo-planar imaging-based sequence with the following parameters: TR = 11,600, TE = 85, FOV = 256 mm ^*^ 256 mm, acquisition matrix = 112^*^112, axial slices = 32, 64 diffusion directions with b = 1,000 s/mm^2^, and an additional image without b = 0 s/mm^2^.

### Data Processing

We used the SPM8 (Statistical Parametric Mapping: http://www.fil.ion.ucl.ac.uk/spm) and DPARSFA (Data Processing Assistant Resting-State: http://www.restfmri.net) on the preprocessing of all the rs-fMRI data (22). The specific preprocessed steps were as follows: (1) the first 10 volumes of the fMRI were removed; (2) we performed the slice timing, head movement correction, and rearrangement; (3) all subjects were excluded if their head motion which was >2.0 mm maximum displacement in any of the x, y, or z directions was >2° (23); (4) all the rs-fMRI data were spatially normalized to the Montreal Neurological Institute (MNI) space using structural image normalization parameters; (5) the smoothing Gaussian kernel of full width at half maximum (FWHM) was 8 mm (24); (6) the 24 head motion parameters, averaged global, white matter signals, and cerebrospinal fluid were processed by nuisance covariates regression (25); (7) removing linear trends; and (8) temporal band-pass filtering (0.01–0.08 Hz) (26).

The DTI data were preprocessed using PANDA (http://www.nitrc.org/projects/panda) (27) in MATLAB2014a. The specific preprocessed steps were as follows: in MATLAB2014a. (1) converting DICOM files into NIfTI images; (2) estimating the brain mask; (3) cropped the image; (4) correction of the eddy current effect and head motions; (5) averaging multiple acquisitions; and (7) FA metrics calculation.

### Construction of Brain Networks

The Human Brainnetome Atlas (246 Atlas) were used to demarcate the nodes and edges of the brain network (28). In each subject, 246 atlas were used to construct brain networks for further graph theory analysis.

The GRETNA (a toolbox for analyzing brain network, www.nitrc.org/projects/gretna/) were used to construct the functional brain network. For each subject, Pearson correlation coefficient was used to construct the correlation matrix which the mean time series of each region was represented each node. Fisher Z transform was applied to each matrix to convert the data into Z-scores. A FA-weighted symmetric matrix was constructed for each participant by deterministic tractography as the following analysis basis. Each matrix represented the white matter network of the cerebral cortex, and each row or column in network represented the brain region of 246 atlas.

### Threshold Calculation

To construct an undirected binary network and make the generated graph metrics stable, it is necessary to be thresholded for the weight of the brain networks. There is no fixed method to determine the threshold in current research. Therefore, in FN, we used sparsity (26–50%) with a step of 1% (29) to divide the network threshold. Then, we calculated the topological properties of FN in a series of threshold range. In SN, we use FA (0.20.42) with a step of 0.2 as the threshold of the network according to the previous study (30). The small-worldness property is related to the threshold of the network (31), so we need to calculate a network threshold to get effective network properties.

### Whole Brain Network Organization

Graph theoretical analyses of the FN and SN in patients with RC and HC were calculated with routines from the GRETNA toolbox. The network topological properties at the global levels were calculated, including (1) properties that suggest network segregation of brain, such as the normalized clustering coefficient (γ), the local efficiency *E*_*loc*_(*G*); (2) properties that indicate network integration of the brain, such as the normalized shortest path length(λ), the global efficiency *E*_*glob*_(*G*); (3) small-worldness (δ) property which evaluates the balance of segregation and integration.

The nodal efficiency (*E*_*nod*_) measures the ability of a particular node to propagate information with all other nodes in the network. It is considered as the inverse of the harmonic mean of the minimum path length between an index node and all other nodes in the network.

### Network Resilience Analysis

Network resilience refers to the ability to withstand perturbations or failures in the network, which is usually related to the stability of complex networks (32, 33). In FN and SN, we used random or targeted attacks with fixed sparsity or FA values to evaluate the network resilience, so as to ensure that all anatomical regions were involved in the network, thus minimizing the number of false-positive paths (32). In targeted attack analysis, the betweenness value of each node in the network was calculated and sorted in descending order. We deleted the nodes in the network in order of betweenness value and calculated the global efficiency of each network after attack (34). In random attack analysis, we deleted the nodes of network randomly and calculated the global efficiency of each network after attack.

### Statistical Analysis

The demographic and clinical characteristics of the patients with RC and HC were analyzed by chi-square test and two-sample *t*-tests using SPSS 21. We performed statistical comparisons of topological measures between the two groups using non-parametric permutation tests with 5,000 iterations for each sparsity and FA value (35). For the *E*_*nod*_, the non-parametric permutation tests was repeated at the sparsity = 26% and the FA value = 0.42. FDR correction was conducted for all these results. Besides, we used Pearson correlation analyses to explore the correlations in patients with RC between nodes with significant difference in *E*_*nod*_ and the severity of depression (HAMD score).

## Results

### Demographic Characteristics

Demographic information is summarized in Table 1. There is no significant difference in age (*p* = 0.564) and gender (χ^2^ =1.312, *p* = 0.765) between the two groups. There was a significant difference in HAMD score between the two groups (*p* < 0.001).

**Table 1:** Demographic and clinical characteristics of subjects.

**Variables (Mean ±SD)**	**Patients with RC** **(*n* = 42)**	**Healthy controls** **(*n* = 38)**	***p*-value**
Gender (M:F)	23:19	22:16	0.564#
Age (years)	50.89 ± 7.50	48.96 ± 7.93	0.756*
HAMD	9.94 ± 4.93	_	_

*SD, standard deviation; HAMD, Hamilton depression rating scale*.

*^#^ and*

^*^ indicate p-value for chi-square test and two-sample t-test, respectively.

### The Alterations of FN and SN Properties

**Figure 1:**
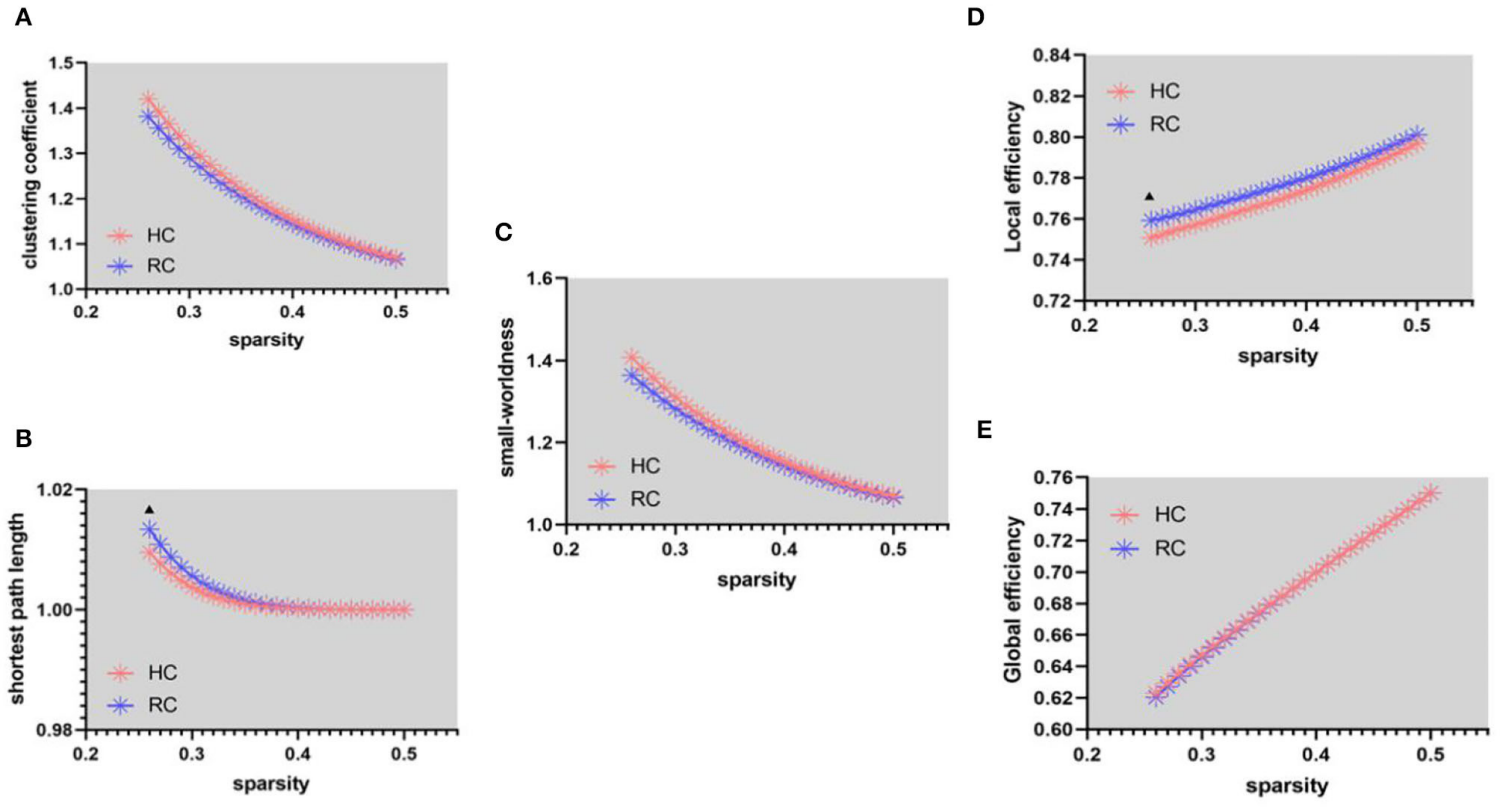
Functional connectivity network at different sparsity for patients with RC (the blue line) and controls (the pink line) and their statistical comparison results (*p* < 0.05 5,000 permutation test, FDR correction). **(A)** Gamma, **(B)** lambda, **(C)** sigma, **(D)** local efficiency, and **(E)** global efficiency. The black triangles indicate a significant group difference.

In the functional network, patients with RC have a higher shortest path length (λ) than HC (Figure 1B, sparsity = 26%). Since there is no significant difference in the clustering coefficient (γ) between the two groups under the same sparsity (Figure 1A), this leads to the abnormal small-worldness(σ) (Figure 1C) of patients with RC. The local efficiency has increased in patients with RC (Figure 1D, sparsity = 26%), and there is no significant change in global efficiency (Figure 1E).

**Figure 2:**
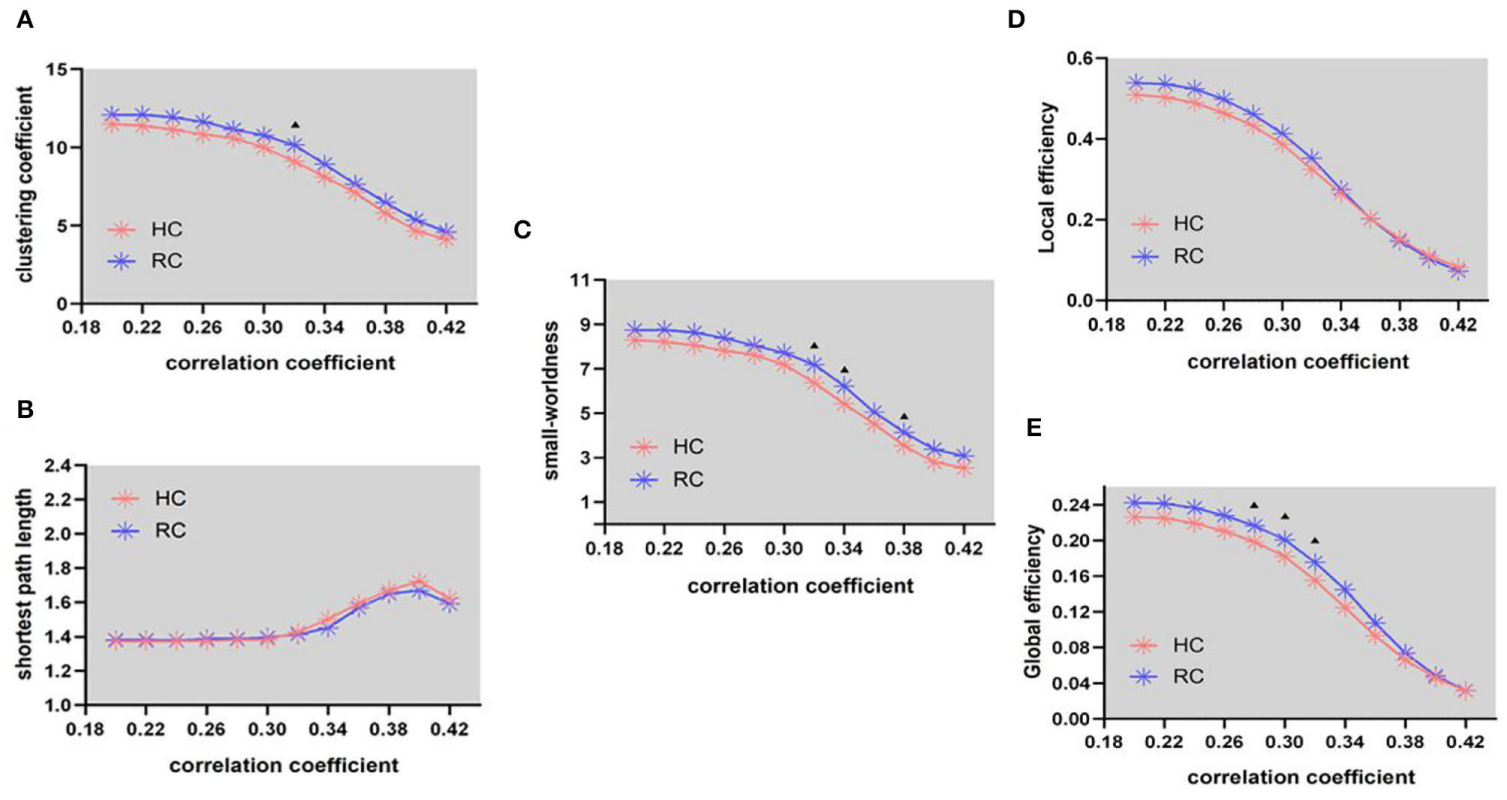
Structural connectivity network at different FA threshold for RC (the blue line) and controls (the pink line) and their statistical comparison results (*p* < 0.05 5000 permutation test, FDR correction). **(A)** Gamma, **(B)** lambda, **(C)** sigma, **(D)** local efficiency, and **(E)** global efficiency. The black triangles indicate a significant group difference.

In the structural network, the clustering coefficient (γ) (Figure 2A, FA = 0.32) and small-worldness(σ) (Figure 2C, FA = 0.32, 0.34, and 0.38) of patients with RC is larger than that of HC, the shortest path length (λ) (Figure 1B) being unchanged. In addition, compared with HC, the global efficiency of patients with RC has increased (Figure 2E, FA = 0.28–0.32).

### Regional Efficiency Comparison

**Figure 3:**
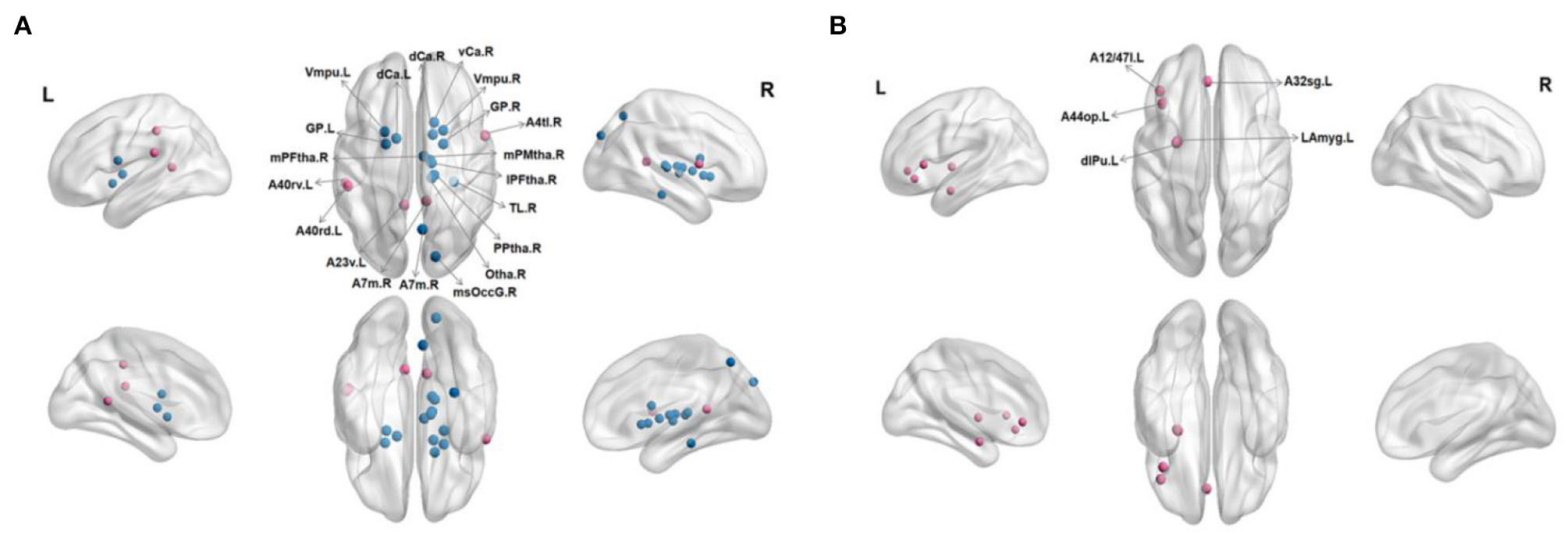
Regions with significant differences in nodal efficiency between patients with RC and HC. Nonparametric permutation tests were applied to nodal efficiency of all 246 cortical regions (*p* < 0.05; 5,000 permutation test, FDR correction). **(A)** Represented FN, and **(B)** represented SN. Red is for increased nodal efficiency in RC patients group, while blue is for decreased nodal efficiency in RC patients group. L, left; R, right.

In FN, patients with RC showed that significantly decreased nodal efficiency. There were several regions including bilateral basal ganglia, right parahippocampal gyrus, bilateral thalamus, right precuneus, and right lateral occipital cortex. Meanwhile, the increased nodal efficiency was mainly in frontal lobe (orbital gyrus), basal ganglia, left inferior frontal gyrus, left amygdala, bilateral cingulate gyrus, left inferior parietal lobule, and right precentral gyrus in FN and SN for patients with RC (*p* < 0.05, after 5,000 permutation test, FDR test) (Tables 2, 3; Figure 3).

**Table 2:** Brain regions with significant group effect in the nodal efficiency between patients with RC and HC for FN.

	**Regions**	**Patients with RC**	**Control**	***p*-value**
**RC** **<** **HC**
	vmPu.R	0.5580 ± 0.0582	0.6190 ± 0.0578	0.0002
	dCa.L	0.5116 ± 0.0694	0.5816 ± 0.0592	0.0002
	GP.R	0.5575 ± 0.0732	0.6195 ± 0.0526	0.0002
	vmPu.L	0.5765 ± 0.0490	0.6264 ± 0.0542	0.0002
	TL.R	0.5874 ± 0.0466	0.5941 ± 0.0533	0.0008
	PPtha.R	0.5245 ± 0.0737	0.5846 ± 0.0519	0.0004
	GP.L	0.5581 ± 0.0661	0.6107 ± 0.0620	0.0006
	lPFtha.R	0.5645 ± 0.0698	0.6148 ± 0.0589	0.0032
	vCa.R	0.5223 ± 0.0507	0.5645 ± 0.0514	0.0044
	A7m.R	0.6101 ± 0.0540	0.6404 ± 0.0656	0.0060
	Otha.R	0.5318 ± 0.0491	0.5833 ± 0.0610	0.0052
	mPMtha.R	0.5719 ± 0.0416	0.6158 ± 0.0542	0.0060
	msOccG.R	0.6029 ± 0.0705	0.6429 ± 0.0569	0.0098
	dCa.R	0.5286 ± 0.0650	0.5701 ± 0.0647	0.0090
	mPFtha.R	0.5545 ± 0.0615	0.6007 ± 0.0778	0.0090
**RC** **>** **HC**
	A23v.L	0.6719 ± 0.0485	0.6601 ± 0.0612	0.0018
	A40rd.L	0.6862 ± 0.0527	0.6738 ± 0.0686	0.0042
	A40rv.L	0.7195 ± 0.0408	0.6875 ± 0.0388	0.0048
	A23v,R	0.6742 ± 0.0394	0.6360 ± 0.0479	0.0064
	A4tl.R	0.7038 ± 0.0857	0.6709 ± 0.0631	0.0048

*vmPu, ventromedial putamen; dCa, dorsal caudate; GP, globus pallidus; TL, area TL (lateral PPHC, posterior parahippocampal gyrus); PPtha, posterior parietal thalamus; lPFtha, lateral prefrontal thalamus; vCa, ventral caudate; A7m, medial area 7(PEp); Otha, occipital thalamus; mPMtha, pre-motor thalamus; msOccG, medial superior occipital gyrus; mPFtha, medial prefrontal thalamus; L, left; R, right*.

**Table 3:** Brain regions with significant group effect in the nodal efficiency between patients with RC and HC for SN.

	**Regions**	**Patients with RC**	**Control**	***p*-value**
**RC** **>** **HC**				
	A12/47l.L	0.2498 ± 0.0352	0.2099 ± 0.0717	0.0036
	dlPu.L	0.3117 ± 0.0386	0.2774 ± 0.0610	0.0036
	A44op.L	0.2461 ± 0.0316	0.2105 ± 0.0698	0.0054
	A32sg.L	0.2365 ± 0.0365	0.1857 ± 0.0993	0.0062
	LAmyg.L	0.2589 ± 0.0269	0.2148 ± 0.0923	0.0086

*A12/47l, lateral area 12/47; dlPu, dorsolateral putamen; A44op, opercular area 44; A32sg, subgenual area 32; LAmyg, lateral amygdala; L, left; R, right*.

### The Analysis of Network Resilience

**Figure 4:**
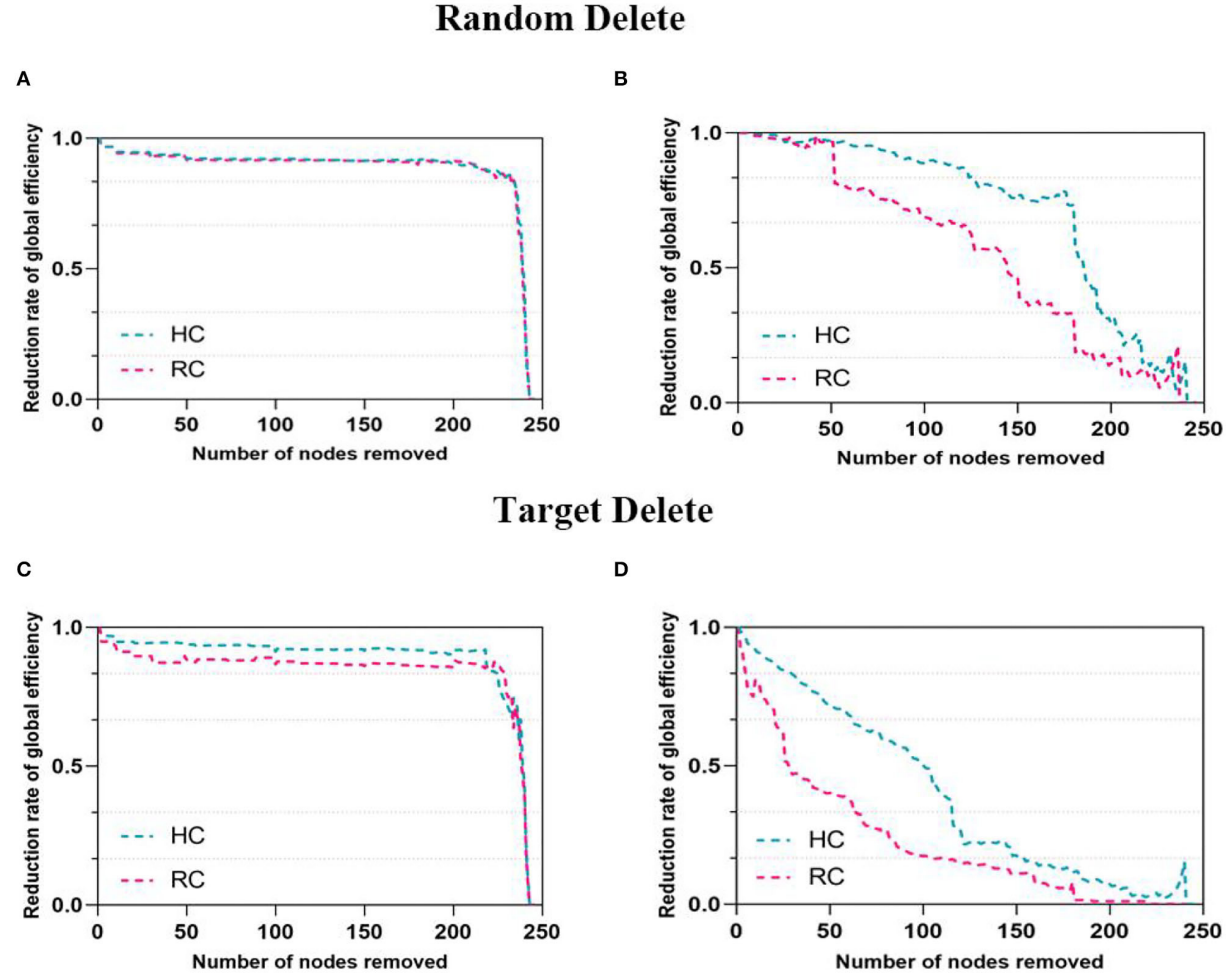
Network resilience under random and target analysis. The alterations of global efficiency under removing node at random (first panel) and targeted pattern (second panel). The blue line corresponded to the performance of HC, pink line for RC. The **(A,C)** were for FN and the **(B,D)** were for SN.

With the targeted and random attack, a significantly decreased decline of the global efficiency was found in FN and SN (Figure 4). In both networks, the global efficiency of patients with RC decreased faster over a wide percentage of removal, which reflected that the networks of patients with RC were more fragile. In all subjects, the resilience of structural network is weaker than that of functional network under the same threshold.

### Correlations Between Network Properties and HAMD Scores for RC

**Figure 5:**
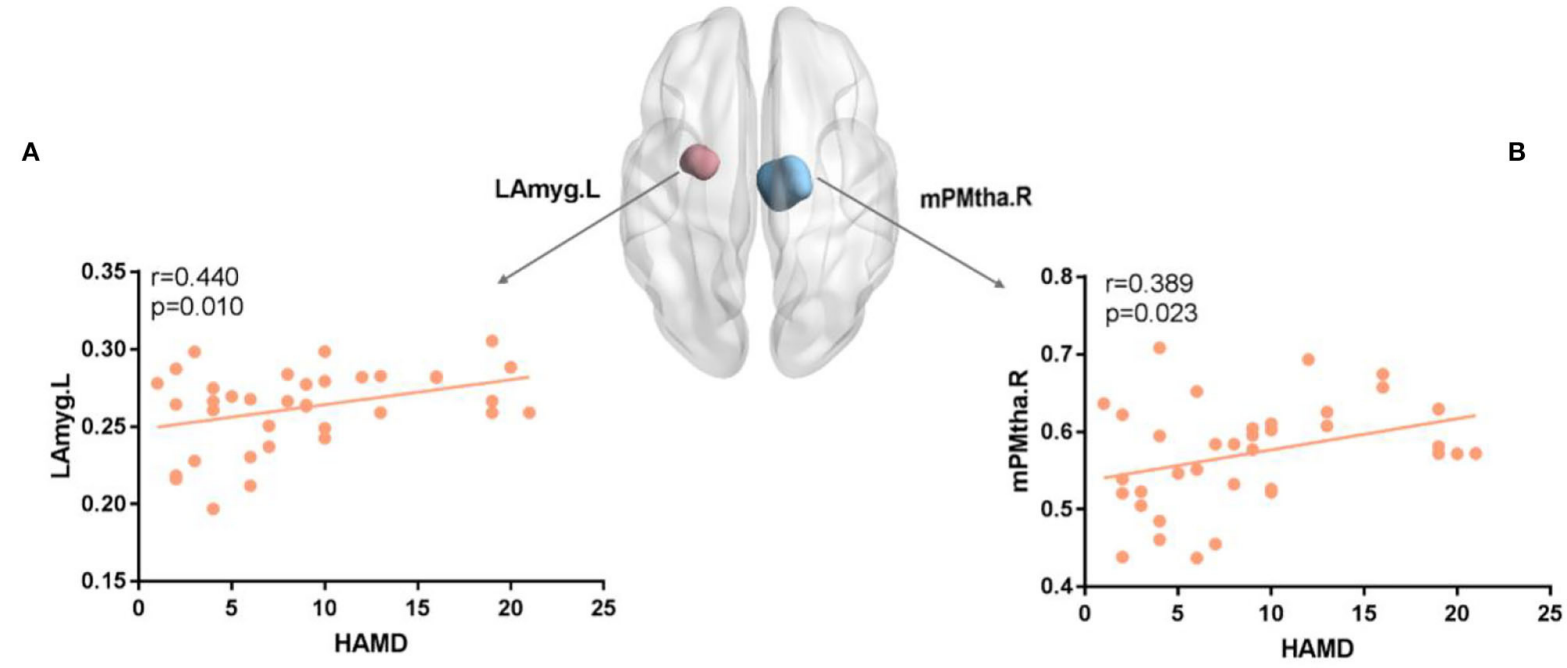
**(A,B)** The nodal efficiency of several regions was positively correlated with the HAMD score for FN and SN. The brain map showed regions with decreased *E*_*nod*_(Blue for mPMtha.R and pink for LAmyg.L). mPFtha, medial prefrontal thalamus; LAmyg, lateral amygdala; L, left; R, right.

In the analysis, there were correlations between the HAMD score and the nodes with significant *E*_*nod*_ in the FN. For FN, mPMtha.R (r = 0.389, *p* = 0.023) (Figure 5B), and for SN, LAmyg.L (r = 0.440, *p* = 0.01) (Figure 5A).

## Discussion

In this study, different topological organizations of FN and SN in patients with RC and HC were explored. The findings pointed patients with RC displayed altered small-worldness property and global topological organization compared with HC. Moreover, there were regions with significant abnormal *E*_*nod*_ being mainly distributed in frontal region, subcortical regions, and central region in patients with RC. In addition, patients with RC showed vulnerable network resilience in both networks, and FN would be more stable than SN across participants.

### Network Properties

Many studies have used fMRI (36, 37) and DTI (21) images to explore the global and regional brain network properties of patients with breast cancer and lung cancer, but there are still few studies on rectal cancer. Compared with HC, the functional networks of patients with RC displayed a higher shortest path length (λ) and decreased small-worldness(σ), reflecting reduced global integration and disrupted organization balance (1, 21). Our results also revealed increased local efficiency in patients with RC. It is a measure of local information transmission among adjacent nodes and therefore an indication of network segregation (38, 39). Previous studies demonstrated reduced local efficiency, a common measure of the brain network's response to computational attack, associated with patients with breast cancer (21, 40). Due to brain structural damage, decreased local efficiency would affect the fault tolerant ability of brain network. More detail, the result of weakening network fault tolerance is that if a node in the brain is damaged, the connection between previously linked nodes would be greatly affected (41). Therefore, reduced local efficiency is a risk factor for patients with RC. Recently, researchers use graph theory to analyze complex brain functional networks after chemotherapy. It has been proved that chemotherapy-related cognitive deficits were associated with abnormal topological alterations of brain functional and structural network (42, 43). In this study, increased shortest path length and decreased local efficiency in patients with RC with surgery and chemotherapy could be seen as a brain compensation mechanism, which included changing the global pathway and adjusting regional activity to preserve a seesaw-like balance of the brain network.

Patients with RC showed increased clustering coefficients (γ), small-worldness(σ), and global efficiency in SN (Figure 2). Abnormal small-worldness property of SN indicated that the local specialization and global integration of brain in patients with RC were disrupted, where the SN tended to be more randomized (44). Global efficiency is the inverse of the average shortest path between nodes. When nodes could interact directly, the efficiency is high (21). Therefore, global efficiency is an indicator of network function integration and parallel information processing capability (41). The present results of abnormal network properties reflected the undesired topological organization in SN, which exhibited that the deficits of emotional and cognitive processing in patients with RC might result from network damages. Besides, the increased network properties of SN in patients with RC might suggest that local nerve fibers reconstructed in response to the abnormalities in brain functional network. The compensatory response of the SN is activated for maintaining brain functional integrity to compensate the cognitive impairment caused by chemotherapy to patients with RC (24). Aforementioned evidence illuminated that cognitive deficit related to patients with RC may act *via* disrupted coordination between global and regional networks.

### Nodal Efficiency of Networks

To explore the functional and structural characteristics of the human brain more accurately and quantitatively, our study employed a new standard brain atlas, containing 246 brain regions. This atlas would allow brain network analysis to use predefined nodes in an informed manner (45). Therefore, more detailed division of brain regions provides better help in multimodal data analysis. We observed decreased *E*_*nod*_ only in FN of patients with RC. The significantly changed regions were located in bilateral basal ganglia, bilateral thalamus, right parahippocampal gyrus, right precuneus, and right lateral occipital cortex. The basal ganglia is not only related to motor control, but also related to the cognitive and limbic functions (45). Moreover, basal ganglia is the collection of subcortical nuclei surrounding the thalamus (46). Abnormal activation of basal ganglia/thalamus was found in the depressive studies (47), suggesting that abnormalities in these brain regions may lead to abnormal emotional processing mechanisms. Prior studies reported that parahippocampal gyrus and precuneus were associated with memory function, so alterations in these regions might affect memory decline (48, 49). Task fMRI study of memory factors found that the occipital cortex of patients with cancer was more significantly correlated with vigor and fatigue scores (50). Frequent fatigue is a common symptom of patients with cancer (51). Aforementioned evidence indicated that the decreased *E*_*nod*_ of FN in this study represented alterations in regional characteristics of the brain network, which further affected the cognitive impairment of patients with RC.

Furthermore, the increased nodal efficiency was mainly in frontal cortex, left amygdala, bilateral cingulate gyrus, left inferior parietal lobule, and right precentral gyrus in FN and SN for patients with RC. In experiments with high-demand condition, the right inferior frontal gyrus and other components of the two hemisphere working memory circuitry in patients with cancer were found greater activation than the control group in a prior study (52). These abnormalities might be a compensation mechanism to preserve normal thinking and responsiveness in patients with cancer. In addition, chemotherapy affects estrogen levels in patients with cancer. Estrogen levels are thought to have neuroprotective effects in the brain, thus helping to maintain cognitive function (53). Therefore, female patients with cancer are more likely to develop cognitive impairment in brain regions related to learning and memory after chemotherapy, such as hippocampus and amygdala (54). The anterior cingulate cortex is involved in attention control, response selection, and error monitoring (55). Abnormal brain activity patterns in the attention-controlled regions, including the anterior cingulate gyrus, are related to anxiety (56). The emotional fluctuation caused by excessive psychological stress in patients with RC could induce abnormal activation of cingulate gyrus. Saykin et al. (57) revealed that the activation of frontal and parietal lobes increased during the speech working memory task 1 month after chemotherapy. Compared with controls, the cancer group showed significantly greater activation in right precentral gyrus and right cingulate gyrus (19). Moreover, in the SN, the nodal efficiency was only increased. We speculated that after surgery and chemotherapy, the node efficiency of SN showed more obvious activation to maintain the robustness of overall network at the expense of other network property, such as integration. These results improved the understanding of chemotherapy-induced cognitive impairment in patients with RC from the perspective of brain node efficiency.

As shown in Figure 5, the patients with RC showed a positive relationship between HAMD and decreased nodal efficiency in mPMtha.R of FN, as well as a positive relationship between HAMD and increased nodal efficiency in LAmyg.L of SN. The correlation between the changed node efficiency and HAMD score may indicate impaired cognitive control combined with abnormal affective processing in patients with RC (29). A prior study suggested that regions sensitive to negative emotions were hyperactive in processing negative information (36), and it was not surprising to find a significant positive correlation between increased nodal efficiency and HAMD in the amygdala. Moreover, the positive relationship between HAMD and decreased nodal efficiency revealed that abnormal activation of FN in patients with RC might cause cognitive impairments and depressed mood (58). Therefore, we speculated that alterations in brain network properties assist us to study the depression tendency in patients with RC after chemotherapy and surgery.

### Comparison of Network Resilience

In both networks, a key finding of significantly decreased resilience to targeted and random attack was found (Figure 4). Being more effective than other network properties to measure network integration performance, global efficiency of the FN and SN was utilized to explore network resilience quantitatively (34). In this study, both networks of patients with RC were more vulnerable and SN is less resilient than FN, which were consistent with our previous research results (59). This finding enhanced the conclusion that lower brain resilience was associated with progressive deterioration of cognitive impairment in breast cancer survivors (21). Similar results were investigated in other neurological diseases such as major depressive disorders (33) and temporal lobe epilepsy (32). A previous study showed that the degree distribution of brain network followed the exponentially truncated power law (60). This exponentially truncated power law distribution may be helpful in resisting the targeted attack of the hubs, which means that the brain networks of two groups were almost constant when deletion rate was low (61). The deletion ratios reaching 50%, and the decline rate of global efficiency in networks began to exhibit obvious differences. Exploring the resilient of networks actually simulated the process of cognitive decline in all participants. In detail, as the important nodes were deleted, the functional and structural integrity of brain networks were impaired. Additionally, the FN was more resilient than the SN this study, which were similar to these findings in the previous studies (30, 62). A prior study discovered that there was commonly a functional connectivity between regions that have no direct structural connectivity, implying that functional network was a more stable system in brain network (63). Therefore, functional networks were more robust to node removal. Our results may provide a new direction for studying cognitive impairment in patients with RC after surgery and chemotherapy.

## Conclusion

This study explores the effects of depression tendency on brain functional and structural network in patients with RC with surgery and chemotherapy through multimodal brain connectivity analysis. Patients with RC show the abnormal small-worldness property and network topological organization in FN and SN. The alterations in nodal parameter are mainly observed in the limbic and parietal lobes as well as the subcortical nuclei in patients with RC. The patients with RC demonstrate significant cognitive impairment compared with HC, and this impairment may be associated with lower network attack tolerance. The discovery of functional and structural networks is critical for understanding the neurobiological mechanism associated with depression tendency in patients with RC with surgery and chemotherapy.

## Limitation

The lack of follow-up data limited the ability of studying the causal relationship between alterations in brain network and depression tendency of patients with RC. The statistical power is restricted by small sample size to some extent. Finally, this study lacks the joint analysis for multimodal data. It is very meaningful to use different modal data for fusion research.

## Data Availability Statement

The raw data supporting the conclusions of this article will be made available by the authors, without undue reservation.

## Ethics Statement

The studies involving human participants were reviewed and approved by Medical Ethics Committee of Gansu Provincial People's Hospital. The patients/participants provided their written informed consent to participate in this study.

## Author Contributions

Conceived and designed the experiments: WZ, YZ, FZ, NM, GH, ZY, and BH. Analyzed the data and wrote the paper: WZ, YY, and YZ. Contributed reagents/materials/analysis tools: WZ, YZ, YL, GH, ZY, and BH. All authors contributed to and have approved the final manuscript.

## Funding

This work was supported in part by the National Key Research and Development Program of China (Grant No. 2019YFA0706200), in part by the National Key Research and Development Program of China (Grant No. 2016YFC1307203), in part by the National Natural Science Foundation of China (Grant Nos. 61632014, 61627808, 62176140, and 82001775), in part by the Natural Science Foundation of Gansu Province of China (Grant No. 20JR5RA292), and in part by the Department of Education of Gansu Province: Innovation Star Project for Excellent Postgraduates (2021CXZX-121), in part by Gansu Provincial Hospital Youth Research Fund Project, 18GSSY5-5, in part by the Natural Science Foundation of Shandong Provincial of China (Grant Nos. ZR2021MH120 and ZR2020MG013), in part by the National Social Science Foundation of China (Grant No. 20BSH151), in part by Special Fund of Shandong Medical Association (YXH2021ZX055), in part by the Doctoral Scientific Research Foundation of Shandong Technology and Business University (Grant No. BS 202016), in part of Horizontal project of Yantai Vocational College (No. HX2021041).

## Conflict of Interest

The authors declare that the research was conducted in the absence of any commercial or financial relationships that could be construed as a potential conflict of interest.

## Publisher's Note

All claims expressed in this article are solely those of the authors and do not necessarily represent those of their affiliated organizations, or those of the publisher, the editors and the reviewers. Any product that may be evaluated in this article, or claim that may be made by its manufacturer, is not guaranteed or endorsed by the publisher.

## References

[1] Bruno J Hosseini SH Kesler S. Altered resting state functional brain network topology in chemotherapy-treated breast cancer survivors. Neurobiol Dis. (2012) 48:329–38. 10.1016/j.nbd.2012.07.009 22820143 PMC3461109

[2] Li M Gu J. Changing patterns of colorectal cancer in China over a period of 20 years. World J Gastroenterol. (2005) 11:4685. 10.3748/wjg.v11.i30.4685 16094710 PMC4615411

[3] Wefel JS Kesler SR Noll KR Schagen SB. Clinical characteristics, pathophysiology, and management of noncentral nervous system cancer-related cognitive impairment in adults. CA Cancer J Clin. (2015) 65:123–38. 10.3322/caac.21258 25483452 PMC4355212

[4] Morean DF O'Dwyer L Cherney LR. Therapies for cognitive deficits associated with chemotherapy for breast cancer: a systematic review of objective outcomes. Arch Phys Med Rehabil. (2015) 96:1880–97. 10.1016/j.apmr.2015.05.012 26026579

[5] Linden W Vodermaier A MacKenzie R Greig D. Anxiety and depression after cancer diagnosis: prevalence rates by cancer type, gender, and age. J Affect Disord. (2012) 141:343–51. 10.1016/j.jad.2012.03.025 22727334

[6] Kesler SR Rao A Blayney DW Oakley-Girvan IA Karuturi M Palesh O. Predicting long-term cognitive outcome following breast cancer with pre-treatment resting state fMRI and random forest machine learning. Front Hum Neurosci. (2017) 11:555. 10.3389/fnhum.2017.00555 29187817 PMC5694825

[7] Zhao F Zhang X Thung K-H Mao N Lee S-W Shen D. Constructing Multi-view High-order Functional Connectivity Networks for Diagnosis of Autism Spectrum Disorder. IEEE Trans Biomed Eng. (2021) 69:1237–50. 10.1109/TBME.2021.3122813 34705632

[8] Billiet T Emsell L Vandenbulcke M Peeters R Christiaens D Leemans A. Recovery from chemotherapy-induced white matter changes in young breast cancer survivors? Brain Imaging Behav. (2018) 12:64–77. 10.1007/s11682-016-9665-8 28102529

[9] Eberling JL Wu C Tong-Turnbeaugh R Jagust WJ. Estrogen-and tamoxifen-associated effects on brain structure and function. Neuroimage. (2004) 21:364–71. 10.1016/j.neuroimage.2003.08.037 14741674

[10] McDonald BC Saykin AJ. Alterations in brain structure related to breast cancer and its treatment: chemotherapy and other considerations. Brain Imaging Behav. (2013) 7:374–87. 10.1007/s11682-013-9256-x 23996156 PMC3869865

[11] Janelsins MC Kohli S Mohile SG Usuki K Ahles TA Morrow GR. An Update on Cancer-and Chemotherapy-Related Cognitive Dysfunction: Current Status, Seminars in Oncology. Elsevier (2011). p. 431–8. 10.1053/j.seminoncol.2011.03.014 PMC3120018 21600374

[12] Vahdaninia M Omidvari S Montazeri A. What do predict anxiety and depression in breast cancer patients? a follow-up study. Soc Psychiatry Psychiatr Epidemiol. (2010) 45:355–61. 10.1007/s00127-009-0068-7 19458878

[13] Plis SM Weisend MP Damaraju E Eichele T Mayer A Clark VP. Effective connectivity analysis of fMRI and MEG data collected under identical paradigms. Comput Biol Med. (2011) 41:1156–65. 10.1016/j.compbiomed.2011.04.011 21592468 PMC3174276

[14] Spisák T Opposits G Kis S Pohubi L Jakab A Puskás S. BrainCON: graph theory based multimodal brain connectivity analysis and visualization software. Eur Cong Radiol. (2013) 2013:2013. 10.1594/ecr2013/C-2588

[15] Li Y Yao Z Yang Y Zhao F Fu Y Zou Y. A study on PHF-Tau network effected by apolipoprotein E4. Am J Alzheimers Dis Other Demen. (2020) 35:1533317520971414. 10.1177/1533317520971414 33258666 PMC10623995

[16] Bullmore ET Bassett DS. Brain graphs: graphical models of the human brain connectome. Ann Rev Clin Psychol. (2011) 7:113–40. 10.1146/annurev-clinpsy-040510-143934 21128784

[17] Sporns O. The human connectome: a complex network. Ann N Y Acad Sci. (2011) 1224:109–25. 10.1111/j.1749-6632.2010.05888.x 21251014

[18] Cimprich B Reuter-Lorenz P Nelson J Clark PM Therrien B Normolle D. Prechemotherapy alterations in brain function in women with breast cancer. J Clin Exp Neuropsychol. (2010) 32:324–31. 10.1080/13803390903032537 19642048

[19] Kesler SR Bennett FC Mahaffey ML Spiegel D. Regional brain activation during verbal declarative memory in metastatic breast cancer. Clin Cancer Res. (2009) 15:6665–73. 10.1158/1078-0432.CCR-09-1227 19843664 PMC2859687

[20] Amidi A Hosseini SH Leemans A Kesler SR Agerbæk M Wu LM. Changes in brain structural networks and cognitive functions in testicular cancer patients receiving cisplatin-based chemotherapy. J Natl Cancer Inst. (2017) 109:djx085. 10.1093/jnci/djx085 29617869

[21] Kesler SR Watson CL Blayney DW. Brain network alterations and vulnerability to simulated neurodegeneration in breast cancer. Neurobiol Aging. (2015) 36:2429–42. 10.1016/j.neurobiolaging.2015.04.015 26004016 PMC4464941

[22] Yan C Zang Y. DPARSF: a MATLAB toolbox for “pipeline” data analysis of resting-state fMRI. Front Syst Neurosci. (2010) 4:13. 10.3389/fnsys.2010.00013 20577591 PMC2889691

[23] Wang J Qiu S Xu Y Liu Z Wen X Hu X. Graph theoretical analysis reveals disrupted topological properties of whole brain functional networks in temporal lobe epilepsy. Clin Neurophysiol. (2014) 125:1744–56. 10.1016/j.clinph.2013.12.120 24686109

[24] Liu F Guo W Liu L Long Z Ma C Xue Z. Abnormal amplitude low-frequency oscillations in medication-naive, first-episode patients with major depressive disorder: a resting-state fMRI study. J Affect Disord. (2013) 146:401–6. 10.1016/j.jad.2012.10.001 23116810

[25] Zeng LL Shen H Liu L Hu D. Unsupervised classification of major depression using functional connectivity MRI. Hum Brain Mapp. (2014) 35:1630–41. 10.1002/hbm.22278 23616377 PMC6869344

[26] Wang L Dai Z Peng H Tan L Ding Y He Z. Overlapping and segregated resting-state functional connectivity in patients with major depressive disorder with and without childhood neglect. Hum Brain Mapp. (2014) 35:1154–66. 10.1002/hbm.22241 23408420 PMC6869506

[27] Cui Z Zhong S Xu P Gong G He Y. PANDA: a pipeline toolbox for analyzing brain diffusion images. Front Hum Neurosci. (2013) 7:42. 10.3389/fnhum.2013.00042 23439846 PMC3578208

[28] Fan L Li H Zhuo J Zhang Y Wang J Chen L. The human brainnetome atlas: a new brain atlas based on connectional architecture. Cerebral cortex. (2016) 26:3508–26. 10.1093/cercor/bhw157 27230218 PMC4961028

[29] Guo H Cheng C Cao X Xiang J Chen J Zhang K. Resting-state functional connectivity abnormalities in first-onset unmedicated depression. Neural Regen Res. (2014) 9:153. 10.4103/1673-5374.125344 25206796 PMC4146162

[30] Jiang W Li J Chen X Ye W Zheng J. Disrupted structural and functional networks and their correlation with alertness in right temporal lobe epilepsy: a graph theory study. Front Neurol. (2017) 8:179. 10.3389/fneur.2017.00179 28515708 PMC5413548

[31] Braun U Plichta MM Esslinger C Sauer C Haddad L Grimm O. Test–retest reliability of resting-state connectivity network characteristics using fMRI and graph theoretical measures. Neuroimage. (2012) 59:1404–12. 10.1016/j.neuroimage.2011.08.044 21888983

[32] Bernhardt BC Chen Z He Y Evans AC Bernasconi N. Graph-theoretical analysis reveals disrupted small-world organization of cortical thickness correlation networks in temporal lobe epilepsy. Cerebral cortex. (2011) 21:2147–57. 10.1093/cercor/bhq291 21330467

[33] Ajilore O Lamar M Leow A Zhang A Yang S Kumar A. Graph theory analysis of cortical-subcortical networks in late-life depression. Am J Geriatric Psychiatry. (2014) 22:195–206. 10.1016/j.jagp.2013.03.005 23831171 PMC3858393

[34] Rubinov M Sporns O. Complex network measures of brain connectivity: uses and interpretations. Neuroimage. (2010) 52:1059–69. 10.1016/j.neuroimage.2009.10.003 19819337

[35] Yao Z Zhang Y Lin L Zhou Y Xu C Jiang T. Abnormal cortical networks in mild cognitive impairment and Alzheimer's disease. PLoS Comput Biol. (2010) 6:e1001006. 10.1371/journal.pcbi.1001006 21124954 PMC2987916

[36] Murray EA Wise SP Drevets WC. Localization of dysfunction in major depressive disorder: prefrontal cortex and amygdala. Biol Psychiatry. (2011) 69:e43–54. 10.1016/j.biopsych.2010.09.041 21111403 PMC3058124

[37] Bromis K Gkiatis K Karanasiou I Matsopoulos G Karavasilis E Papathanasiou M. Altered brain functional connectivity in small-cell lung Cancer patients after chemotherapy treatment: a resting-state fMRI study. Comput Math Methods Med. (2017) 2017:1403940. 10.1155/2017/1403940 28798808 PMC5535744

[38] Yu M Linn KA Cook PA Phillips ML McInnis M Fava M. Statistical harmonization corrects site effects in functional connectivity measurements from multi-site fMRI data. Hum Brain Mapp. (2018) 39:4213–27. 10.1002/hbm.24241 29962049 PMC6179920

[39] Zhao F Chen Z Rekik I Lee S-W Shen D. Diagnosis of autism spectrum disorder using central-moment features from low-and high-order dynamic resting-state functional connectivity networks. Front Neurosci. (2020) 14:258. 10.3389/fnins.2020.00258 32410930 PMC7198826

[40] Santarnecchi E Rossi S Rossi A. The smarter, the stronger: intelligence level correlates with brain resilience to systematic insults. Cortex. (2015) 64:293–309. 10.1016/j.cortex.2014.11.005 25569764

[41] Latora V Marchiori M. Efficient behavior of small-world networks. Phys Rev Lett. (2001) 87:198701. 10.1103/PhysRevLett.87.198701 11690461

[42] Deprez S Billiet T Sunaert S Leemans A. Diffusion tensor MRI of chemotherapy-induced cognitive impairment in non-CNS cancer patients: a review. Brain Imaging Behav. (2013) 7:409–35. 10.1007/s11682-012-9220-1 23329357

[43] Kesler SR Adams M Packer M Rao V Henneghan AM Blayney DW. Disrupted brain network functional dynamics and hyper-correlation of structural and functional connectome topology in patients with breast cancer prior to treatment. Brain Behav. (2017) 7:e00643. 10.1002/brb3.643 28293478 PMC5346525

[44] Zhang J Wang J Wu Q Kuang W Huang X He Y. Disrupted brain connectivity networks in drug-naive, first-episode major depressive disorder. Biol Psychiatry. (2011) 70:334–42. 10.1016/j.biopsych.2011.05.018 21791259

[45] de Reus MA Van den Heuvel MP. The parcellation-based connectome: limitations and extensions. Neuroimage. (2013) 80:397–404. 10.1016/j.neuroimage.2013.03.053 23558097

[46] Jaworska N Yang X-R Knott V MacQueen G. A review of fMRI studies during visual emotive processing in major depressive disorder. World J Biol Psychiatry. (2015) 16:448–71. 10.3109/15622975.2014.885659 24635551

[47] Haroon E Fleischer C Felger J Chen X Woolwine B Patel T. Conceptual convergence: increased inflammation is associated with increased basal ganglia glutamate in patients with major depression. Mol Psychiatry. (2016) 21:1351. 10.1038/mp.2015.206 26754953 PMC4940313

[48] Ploner CJ Gaymard BM Rivaud-Péchoux S Baulac M Clémenceau S Samson S. Lesions affecting the parahippocampal cortex yield spatial memory deficits in humans. Cerebral Cortex. (2000) 10:1211–6. 10.1093/cercor/10.12.1211 11073870

[49] Shipman SL Astur RS. Factors affecting the hippocampal BOLD response during spatial memory. Behav Brain Res. (2008) 187:433–41. 10.1016/j.bbr.2007.10.014 18055028

[50] Zunini RAL Scherling C Wallis N Collins B MacKenzie J Bielajew C. Differences in verbal memory retrieval in breast cancer chemotherapy patients compared to healthy controls: a prospective fMRI study. Brain Imaging Behav. (2013) 7:460–77. 10.1007/s11682-012-9213-0 23242968

[51] Servaes P Verhagen C Bleijenberg G. Fatigue in cancer patients during and after treatment: prevalence, correlates and interventions. Eur J Cancer. (2002) 38:27–43. 10.1016/S0959-8049(01)00332-X 11750837

[52] Scherling CS Collins B MacKenzie J Bielajew C Smith A. Pre-chemotherapy differences in visuospatial working memory in breast cancer patients compared to controls: an FMRI study. Front Hum Neurosci. (2011) 5:122. 10.3389/fnhum.2011.00122 22053153 PMC3205481

[53] Goodwin PJ Ennis M Pritchard KI Trudeau M Hood N. Risk of menopause during the first year after breast cancer diagnosis. J Clin Oncol. (1999) 17:2365. 10.1200/JCO.1999.17.8.2365 10561298

[54] McEwen B. Estrogen actions throughout the brain. Recent Prog Horm Res. (2002) 57:357–84. 10.1210/rp.57.1.357 12017552

[55] Devinsky O Morrell MJ Vogt BA. Contributions of anterior cingulate cortex to behaviour. Brain. (1995) 118:279–306. 10.1093/brain/118.1.279 7895011

[56] Engels AS Heller W Spielberg JM Warren SL Sutton BP Banich MT. Co-occurring anxiety influences patterns of brain activity in depression. Cogn Affect Behav Neurosci. (2010) 10:141–56. 10.3758/CABN.10.1.141 20233962 PMC4403735

[57] Saykin A McDonald B Ahles T Chesnut L Wang P Furstenberg C. Altered brain activation following systemic chemotherapy for breast cancer: interim analysis from a prospective fMRI study. In: Abstract presented at 34th Annual Meeting of the International Neuropsychological Society. Boston (2006).

[58] Bürger C Redlich R Grotegerd D Meinert S Dohm K Schneider I. Differential abnormal pattern of anterior cingulate gyrus activation in unipolar and bipolar depression: an fMRI and pattern classification approach. Neuropsychopharmacology. (2017) 42:1399. 10.1038/npp.2017.36 28205606 PMC5436122

[59] Yao Z Zou Y Zheng W Zhang Z Li Y Yu Y. Structural alterations of the brain preceded functional alterations in major depressive disorder patients: evidence from multimodal connectivity. J Affect Disord. (2019) 253:107–17. 10.1016/j.jad.2019.04.064 31035211

[60] Joyce KE Hayasaka S Laurienti PJ. The human functional brain network demonstrates structural and dynamical resilience to targeted attack. PLoS Comput Biol. (2013) 9:e1002885. 10.1371/journal.pcbi.1002885 23358557 PMC3554573

[61] Friedman EJ Landsberg AS. Hierarchical networks, power laws, and neuronal avalanches. Chaos. (2013) 23:013135. 10.1063/1.4793782 23556972 PMC3606226

[62] Damoiseaux JS Rombouts S Barkhof F Scheltens P Stam CJ Smith SM. Consistent resting-state networks across healthy subjects. Proc Nat Acad Sci. (2006) 103:13848–53. 10.1073/pnas.0601417103 16945915 PMC1564249

[63] Honey C Sporns O Cammoun L Gigandet X Thiran J-P Meuli R. Predicting human resting-state functional connectivity from structural connectivity. Proc Nat Acad Sci. (2009) 106:2035–40. 10.1073/pnas.0811168106 19188601 PMC2634800

